# Subtyping non-small cell lung cancer by histology-guided spatial metabolomics

**DOI:** 10.1007/s00432-021-03834-w

**Published:** 2021-11-28

**Authors:** Judith Martha Neumann, Hinrich Freitag, Jasmin Saskia Hartmann, Karsten Niehaus, Michail Galanis, Martin Griesshammer, Udo Kellner, Hanna Bednarz

**Affiliations:** 1 Faculty of Biology, Proteome and Metabolome Research, Center for Biotechnology (CeBiTec), Bielefeld University, Bielefeld, Germany; 2 Institut für Pathologie, Medizinische Hochschule Hannover, Hannover, Germany; 3 Universitätsklinik für Allgemeinchirurgie, Viszeral-, Thorax- und Endokrine Chirurgie, Johannes Wesling Klinikum Minden, Minden, Germany; 4 Universitätsklinik für Hämatologie, Onkologie, Hämostaseologie und Palliativmedizin, Universitätszentrum Innere Medizin, Johannes Wesling Klinikum Minden, Minden, Germany; 5 Institut für Pathologie, Johannes Wesling Klinikum, Minden, Germany; 6 Medical School OWL, AG1: Sustainable Environmental Health Sciences, Bielefeld University, Bielefeld, Germany; 7 Present Address: Clinic for Thoracic Surgery and Thoracic Endoscopy, University Hospital Bielefeld Mitte, Bielefeld, Germany

**Keywords:** Non-small cell lung cancer, Adenocarcinoma, Squamous cell carcinoma, Isocitrate dehydrogenase, Mass spectrometry imaging, Metabolomics

## Abstract

**Purpose:**

Most cancer-related deaths worldwide are associated with lung cancer. Subtyping of non-small cell lung cancer (NSCLC) into adenocarcinoma (AC) and squamous cell carcinoma (SqCC) is of importance, as therapy regimes differ. However, conventional staining and immunohistochemistry have their limitations. Therefore, a spatial metabolomics approach was aimed to detect differences between subtypes and to discriminate tumor and stroma regions in tissues.

**Methods:**

Fresh-frozen NSCLC tissues (*n* = 35) were analyzed by matrix-assisted laser desorption/ionization-mass spectrometry imaging (MALDI-MSI) of small molecules (< m/z 1000). Measured samples were subsequently stained and histopathologically examined. A differentiation of subtypes and a discrimination of tumor and stroma regions was performed by receiver operating characteristic analysis and machine learning algorithms.

**Results:**

Histology-guided spatial metabolomics revealed differences between AC and SqCC and between NSCLC tumor and tumor microenvironment. A diagnostic ability of 0.95 was achieved for the discrimination of AC and SqCC. Metabolomic contrast to the tumor microenvironment was revealed with an area under the curve of 0.96 due to differences in phospholipid profile. Furthermore, the detection of NSCLC with rarely arising mutations of the isocitrate dehydrogenase (IDH) gene was demonstrated through 45 times enhanced oncometabolite levels.

**Conclusion:**

MALDI-MSI of small molecules can contribute to NSCLC subtyping. Measurements can be performed intraoperatively on a single tissue section to support currently available approaches. Moreover, the technique can be beneficial in screening of *IDH*-mutants for the characterization of these seldom cases promoting the development of treatment strategies.

**Supplementary Information:**

The online version contains supplementary material available at 10.1007/s00432-021-03834-w.

## Introduction

Lung cancer is the second most common cancer disease with an incidence of 11.4% and the leading cause of cancer mortality, responsible for 18% of cancer deaths (Sung et al. 2021). The vast majority of lung cancers (85%) belongs to the histologic category of non-small cell lung cancer (NSCLC) (Schabath and Cote 2019). The prevalence of various genetic mutations in NSCLC is described, such as driver mutations in *KRAS* and *epidermal growth factor receptor* (*EGFR*) which occur frequently and can influence therapeutic strategies (Zhu et al. 2017). Within the group of NSCLC, the two most common histological subtypes are the adenocarcinoma (AC) with an occurrence of 40% and the squamous cell carcinoma (SqCC) with an occurrence of 25% (Schabath and Cote 2019). Distinguishing these subtypes is of importance as AC and SqCC have different characteristics and outcomes and therapy regimens differ (Mukhopadhyay and Katzenstein 2011; Wang et al. 2020). However, accurate diagnostic is not always possible via pathological examination of hematoxylin and eosin (HE)-stained slides (Mukhopadhyay and Katzenstein 2011; Osmani et al. 2018). While immunohistochemistry markers work well in 60–100% of the cases, long test duration, difficulties in interpreting the results, and a high tissue consume when testing individual markers are the downsides of this technique (Osmani et al. 2018).

As new diagnostic tools are needed, the aim of this study was to develop an approach to distinguish NSCLC subtypes and to unravel metabolic differences in the tumor microenvironment using matrix-assisted laser desorption/ionization-mass spectrometry imaging (MALDI-MSI)-based metabolomics. This emerging technique allows the label-free analysis of hundreds of endogenous compounds in situ, thus enabling a correlation with histological features (Aichler and Walch 2015; Schwamborn and Caprioli 2010). Due to homogenization of tissues in sample preparation for commonly used mass spectrometry techniques in metabolomics, such as gas chromatography–mass spectrometry and liquid chromatography–mass spectrometry, spatial information is lost (Aichler and Walch 2015). The combination of MALDI-MSI with histopathological analysis can avoid artefacts in data analysis due to varying ratios of healthy and cancer cells within the sections or due to tumor heterogeneity (Schwamborn 2017) and allows for a detailed analysis even of small histologic areas. MALDI-MSI-based metabolomics was successfully applied to reveal differences between NSCLC tumors and normal lung regions based on lipid analysis (Guo et al. 2014; Jones et al. 2014; Lee et al. 2012; Muranishi et al. 2019). Furthermore, lipid MALDI-MSI was used to verify differences between NSCLC subtypes (Lee et al. 2012). These results demonstrate the suitability of MALDI-MSI for small molecule analysis in lung cancer research to explore the metabolism, which is known to be altered in cancers (Pavlova and Thompson 2016). However, the classification of SqCC and AC using MALDI-MSI-based metabolomics and the differences in non-lipid metabolites seem not to be reported yet using this technique.

We, therefore, targeted a classification of AC and SqCC via histology-guided MALDI-MSI based on small molecules by utilizing machine learning algorithms. Additionally, alterations between NSCLC tumor and stroma regions were examined to reveal differences in the tumor microenvironment. Furthermore, it was screened for rare *isocitrate dehydrogenase* (*IDH*)-mutated NSCLC by evaluating oncometabolite signal intensities.

## Materials and methods

### Human material

Lung cancer samples were collected between 2020 and 2021 at the Johannes Wesling Klinikum Minden in Minden, Germany. The study was approved by the Klinisches Ethikkomitee des HDZ NRW (AZ-2019-565). Patients gave informed consent about the usage of resected material.

The study cohort comprises 35 NSCLC samples, consisting of 24 AC (69%) and 11 SqCC (31%). Diagnoses were made by two experienced pathologists on formalin-fixed paraffin-embedded (FFPE) material using HE staining and immunohistochemistry, where necessary. The gain-of-function mutation of the *IDH1* gene was characterized based on DNA extraction from subsequent sections on Illumina MiSeq platform. Detailed information on diagnoses is given in Table 1. Fresh-frozen material was snap-frozen and stored in liquid nitrogen until further use.

**Table 1:** Information on lung cancer specimen used in this study

	AC	SqCC	Total
Cases	24	11	35
Age (median)	68.5	70	69
pT (%)			
1a	2 (8.3)	1 (9.1)	3 (8.6)
1b	3 (12.5)	0 (0)	3 (8.6)
1c	7 (29.2)	1 (9.1)	8 (22.9)
2a	4 (16.7)	2 (18.2)	6 (17.1)
2b	1 (4.2)	2 (18.2)	3 (8.6)
3	4 (16.7)	3 (27.3)	7 (20)
4	2 (8.3)	2 (18.2)	4 (11.4)
n/a	1 (4.2)	0 (0)	1 (2.9)
pN (%)			
0	10 (41.7)	7 (63.6)	17 (48.6)
1	9 (37.5)	4 (36.4)	13 (37.1)
2	2 (8.3)	0 (0)	2 (5.7)
n/a	3 (12.5)	0 (0)	3 (8.6)
*V* (%)			
0	21 (87.5)	9 (81.8)	30 (85.7)
1	2 (8.3)	2 (18.2)	4 (11.4)
n/a	1 (4.2)	0 (0)	1 (2.9)
*L* (%)			
0	18 (75)	8 (72.7)	26 (74.3)
1	5 (20.8)	3 (27.3)	8 (22.9)
n/a	1 (4.2)	0 (0)	1 (2.9)
Stage (%)			
IA	8 (33.3)	2 (18.2)	10 (28.6)
IB	2 (8.3)	1 (9.1)	3 (8.6)
IIA	1 (4.2)	0 (0)	1 (2.9)
IIB	8 (33.3)	6 (54.5)	14 (40)
IIIA	4 (16.7)	2 (18.2)	6 (17.1)
IIIB	1 (4.2)	0 (0)	1 (2.9)
Annotation (%)			
Tumor	24 (100)	11 (100)	35 (100)
Stroma	17 (70.8)	10 (90.9)	27 (77.1)

The tumor node metastasis (TNM) system was used for tumor grading

*NSCLC* non-small cell lung cancer, *SqCC* squamous cell carcinoma, *AC* adenocarcinoma

### Sample preparation

Fresh-frozen tissue samples were sectioned at a thickness of 10 µm in a cryostat, after equilibrating to a temperature of − 20 °C. Sections of AC and SCC specimen were randomly placed onto conductive indium tin oxide (ITO) coated IntelliSlides (Bruker Daltonik GmbH, Germany) and dried under vacuum.

Matrix *N*-(1-naphthyl) ethylenediamine dihydrochloride (NEDC) (≥ 99% p. a., Carl Roth GmbH + Co. KG, Germany) was applied onto ITO slides at a concentration of 7 mg/ml NEDC in methanol/water (70/30, v/v) using a TM-Sprayer (HTX Technologies, LLC, USA). Spraying parameters were kept as following for 28 passes: 0.12 ml/min flow rate, 1200 mm/min velocity, 3 mm track spacing, and a nozzle temperature of 70 °C. Samples were stored in a dry cabinet until measurements.

### MALDI-MSI measurements

Tissue specimen (*n* = 35) were analyzed on a rapifleX MALDI Tissuetyper (Bruker Daltonik GmbH, Bremen, Germany). Samples coated with NEDC were measured in negative reflector mode acquiring spectra between m/z 80–1000. The smartbeam™ 3D laser mode was set to M5 small for the imaging 50 µm application. Spectra were collected with 300 shots per pixel with a frequency of 10,000 Hz. The pulsed ion extraction time was set to 100 ns. The detector gain was regularly adjusted according to the detector check recommendations. Calibration was performed with red phosphorus clusters.

Additionally, AC (*n* = 2) and SqCC (*n* = 2) were analyzed on a Spectroglyph MALDI/ESI Injector (Spectroglyph, LLC, USA) coupled with a Q Exactive Plus mass spectrometer (Thermo Fisher Scientific Inc., USA) for the annotation of detected analytes. Furthermore, the *IDH*-mutated AC was analyzed to confirm metabolite 2-hydroxyglutarate by accurate mass and MS/MS fragments. Measurements were performed in negative mode within a mass range of m/z 85–1000. The laser step size was set to 50 µm with a velocity of 1 mm/s. Mass resolution was set to 70,000 and the inject time was fixed at 250 ms per scan. MS/MS was performed on an isolation window of ± 0.2 m/z with a normalized collision energy of 50 and a maximum inject time of 2000 ms. Pierce Negative Ion Calibration Solution (Thermo Fisher Scientific Inc., USA) was used as external calibrant and matrix peak as internal mass calibrant.

### Histology

After MALDI-MSI measurements, matrix was removed from tissues by washing in 100% methanol for 2 min. Tissues were stained with hematoxylin and eosin (HE) for pathological evaluation. Whole slide scanning was performed on HE sections for digital evaluation of the tissues using a Ventana DP 200 slide scanner (Roche Diagnostics International AG, Rotkreuz, Switzerland). Representative tumor and stroma regions were annotated on whole slide images using the software QuPath (Bankhead et al. 2017) and regions were exported for further analysis using the SCiLS Lab export function (Bruker Daltonik GmbH, Bremen, Germany).

### Data analysis

MSI data were analyzed using the Software SCiLS Lab MVS Pro 2021b (Bruker Daltonik GmbH, Bremen, Germany). Spectra were normalized to the total ion count (TIC) and images were created within a mass range of 0.04 Da. Peaks were manually picked to avoid artefacts and to exclude matrix signals. TIC-normalized intensities for picked peaks (*n* = 137) within annotated regions were imported into Python 3.7 using the SCiLS Python API. Feature importance was applied using a random forest classifier and a threshold of 0.01, leading to 33 ion channels for the analysis of NSCLC subtypes and 23 ion channels for the discriminant analysis of tumor and stroma while excluding the *IDH*-mutated case. Peak lists can be found in the supplementary material. A receiver operating characteristic analysis was implemented to analyze the diagnostic ability of a random forest algorithm and a support-vector machine algorithm using scikit-learn with tenfold cross-validation. Orthogonal partial least squares discriminant analysis (OPLS-DA) was performed using the pypls package and fivefold cross-validation.

High-resolution MALDI-Orbitrap-MSI data were exported to imzML format using ImageInsight (Spectroglyph, LLC, USA) and loaded into SCiLS Lab MVS Pro 2021b (Bruker Daltonik GmbH, Bremen, Germany). Analytes were annotated based on the accurate mass and identified on MS/MS spectra if available using the database METLIN (Smith et al. 2005) with a threshold of 10 PPM.

## Results

### Classification of tissue specimen

Thirty-five human NSCLC cases were used for analyses. The cohort comprises 24 AC (69%) and 11 SqCC (31%). Median patient age at the time of resection was 69. The majority of cases was graded as stage II including 9 AC (37.5%) and 6 SqCC (54.5%), followed by stage I (41.7% AC, 27.3% SqCC) and stage III (20.8% AC, 18.2% SqCC). One specimen harbored a c.395G>T; p.R132L mutation in the isocitrate dehydrogenase gene *IDH1*. The patient presented with stage IIB AC showed high PD-L1 expression of > 90% and mutations of *KRAS* (c.35G>T; p.G12V) and *TP53* (c.797G>T; p.G266V). While tumor areas were present in all cases, stroma regions were annotated in 77.1% of the samples (70.8% AC, 90.9% SqCC). Further information on human material is given in Table 1.

### Classification of NSCLC using MALDI-MSI-based metabolomics and machine learning

Machine learning approaches were used to classify (i) NSCLC tumor (*n* = 34) and NSCLC stroma (*n* = 27) regions as well as (ii) AC tumor (*n* = 23) and SqCC tumor (*n* = 11) regions. The *IDH*-mutated AC was excluded from all analyses, as metabolic alterations were estimated.

**Fig. 1:**
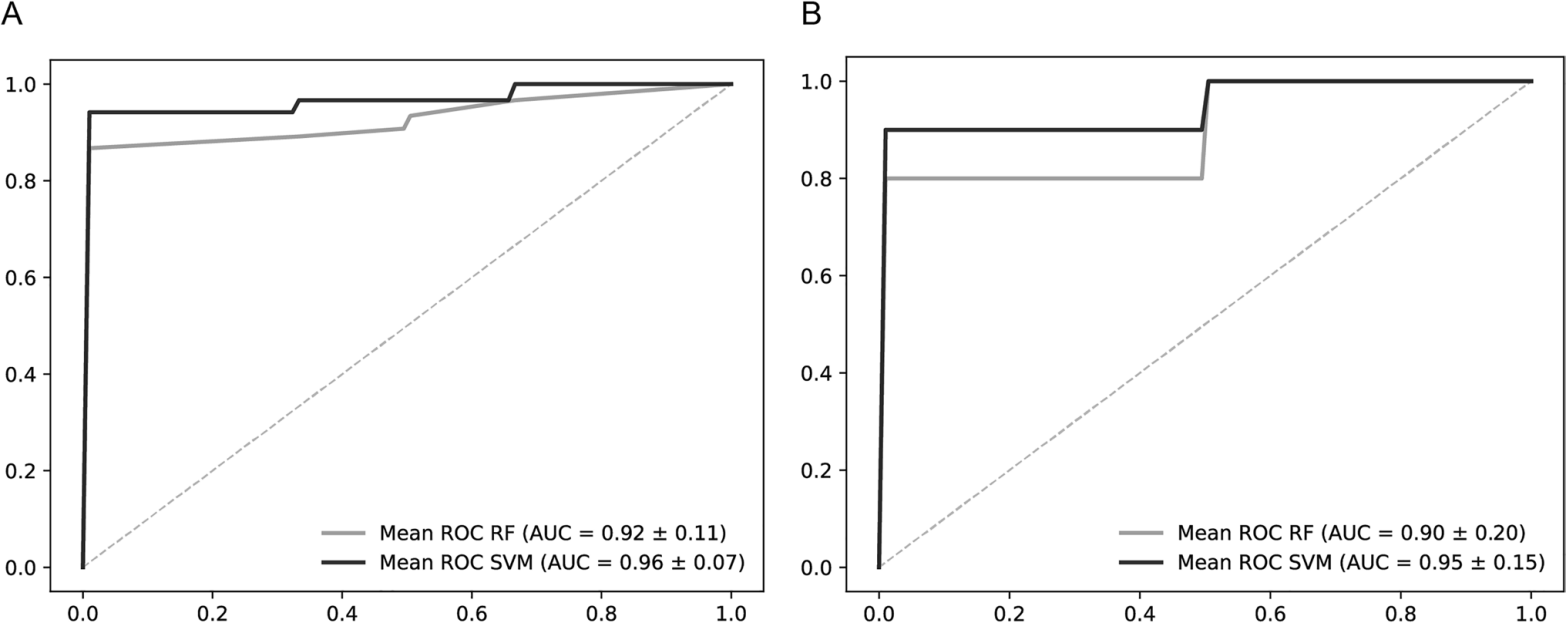
Receiver operating characteristic analyses reveal information on diagnostic ability. Random forest (RF) (grey) and support-vector machine (SVM) (black) algorithms were utilized. **A** Discrimination of NSCLC tumor (*n* = 34) and stroma (*n* = 27). **B** Classification of AC (*n* = 23) and SqCC (*n* = 11). AUC and standard deviation is given. *NSCLC* non-small cell lung cancer, *SqCC* squamous cell carcinoma, *AC* adenocarcinoma, *RF* random forest, *SVM* support-vector-machine

The ability of a random forest (RF) algorithm and of a support-vector machine (SVM) algorithm to distinguish these tissue regions is demonstrated in a receiver operating characteristic analysis (Fig. 1). An area under the curve (AUC) of 0.92 was achieved for the discrimination of tumor and stroma regions using the RF algorithm. The AUC with a SVM classifier was 0.96. Best classification result in the diagnosis of AC tumor and SqCC tumor was also achieved by the SVM algorithm, yielding an AUC of 0.95. AUC with RF algorithm was 0.9. Furthermore, using these algorithms to distinguish AC stroma from SqCC stroma resulted in lower diagnostic ability, indicating higher similarities of stroma metabolome than of tumor metabolome between subtypes (Supplementary Information).

### Uncovering metabolic differences in NSCLC

**Fig. 2:**
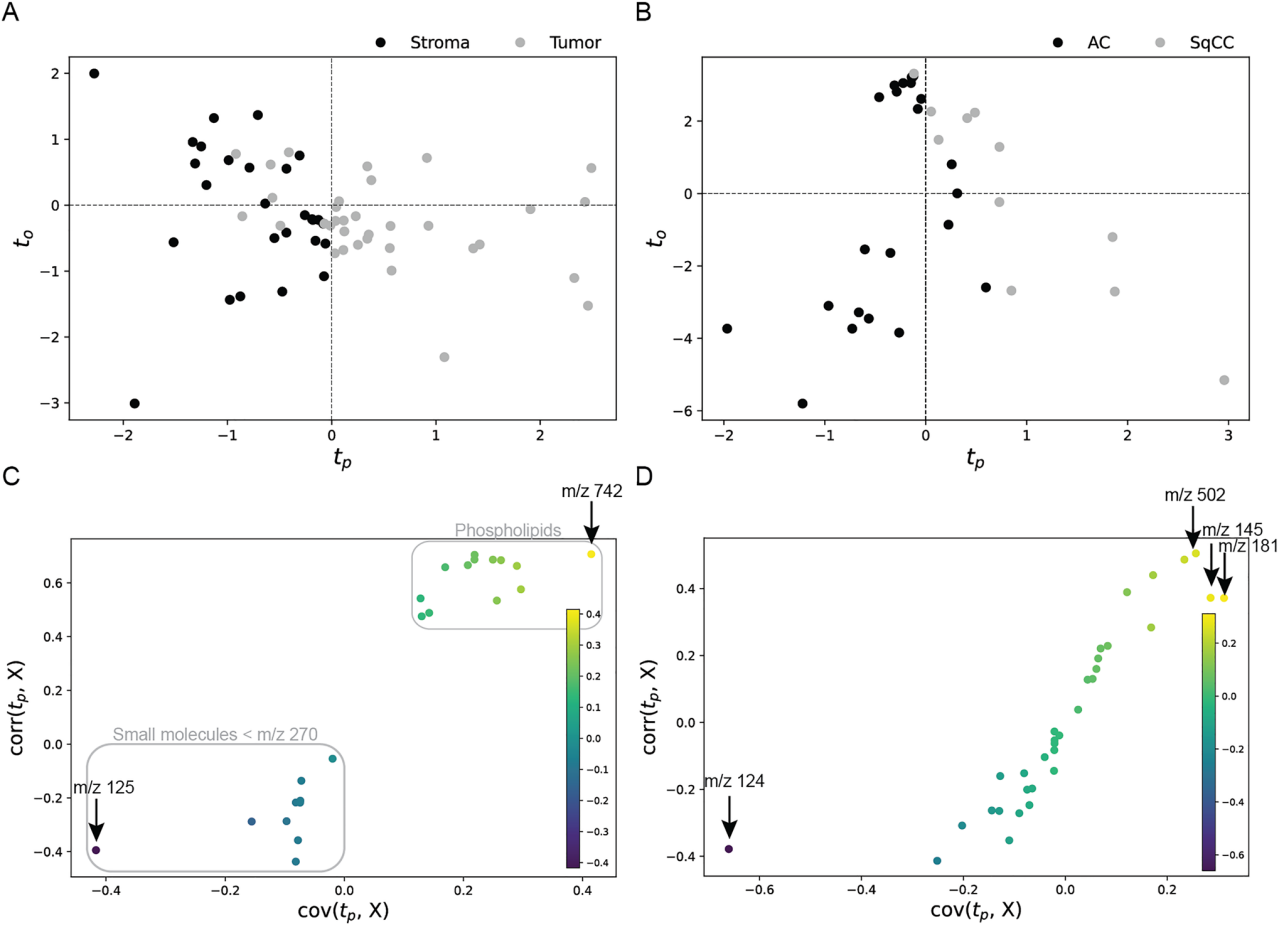
Orthogonal partial least squares discriminant analysis (OPLS-DA) and corresponding S-plots. Analytes with highest contribution to the separation are marked with arrows and corresponding m/z value. **A** OPLS-DA of NSCLC tumor (*n* = 34) and stroma (*n* = 27). **B** OPLS-DA of AC (*n* = 23) and SqCC (*n* = 11). **C** S-plot of the discrimination of NSCLC tumor (*n* = 34) and stroma (*n* = 27) with each of the 23 used peaks visualized as a dot. Small molecules are more prominent in stroma regions and putative phospholipid intensities are higher in tumor regions. **D** S-plot of the discrimination of AC (*n* = 23) and SqCC (*n* = 11) with each of the 33 used peaks visualized as a dot

Multivariate analysis was performed to visualize the discrimination of NSCLC tumor and stroma and of the NSCLC subtypes on metabolite data from MALDI-MSI experiments. The orthogonal partial least squares discriminant analysis (OPLS-DA) shows a discrimination of the groups with minor overlaps (Fig. 2A, B). S-plots were generated to identify analytes strongly contributing to the separation of the groups. The ion channel that is most prominent in tumor regions compared to stroma regions is phospholipid m/z 742 (Fig. 2C). On the other hand, m/z 125 is more prominent in stroma regions (Fig. 2C). Due to overlapping signals with a taurine isotope, an identification via MS/MS was not feasible. Hence, m/z 125 was putatively annotated as the [M + Cl]^−^ ion of oxalic acid based on accurate mass and isotopic pattern. Interestingly, small molecules (m/z < 270) are more prominent in stroma regions, while putative phospholipids contribute to the discrimination of histological regions with high intensities in tumor areas.

In the discriminant analysis of NSCLC subtypes, m/z 124 and m/z 181 show highest differences in AC and SqCC (Fig. 2D). The ion channel m/z 124 is more abundant in AC and was identified as antioxidant taurine. The m/z 181 was annotated as the chloride adduct of analyte glutamine. Supporting this result, the identified [M − H]^−^ ion of glutamine (m/z 145) shows a similar localization in the S-plot and a similar distribution in tissues. Furthermore, m/z 502 contributes to the discrimination with higher intensities in SqCC and was annotated as phosphatidylserine.

**Fig. 3:**
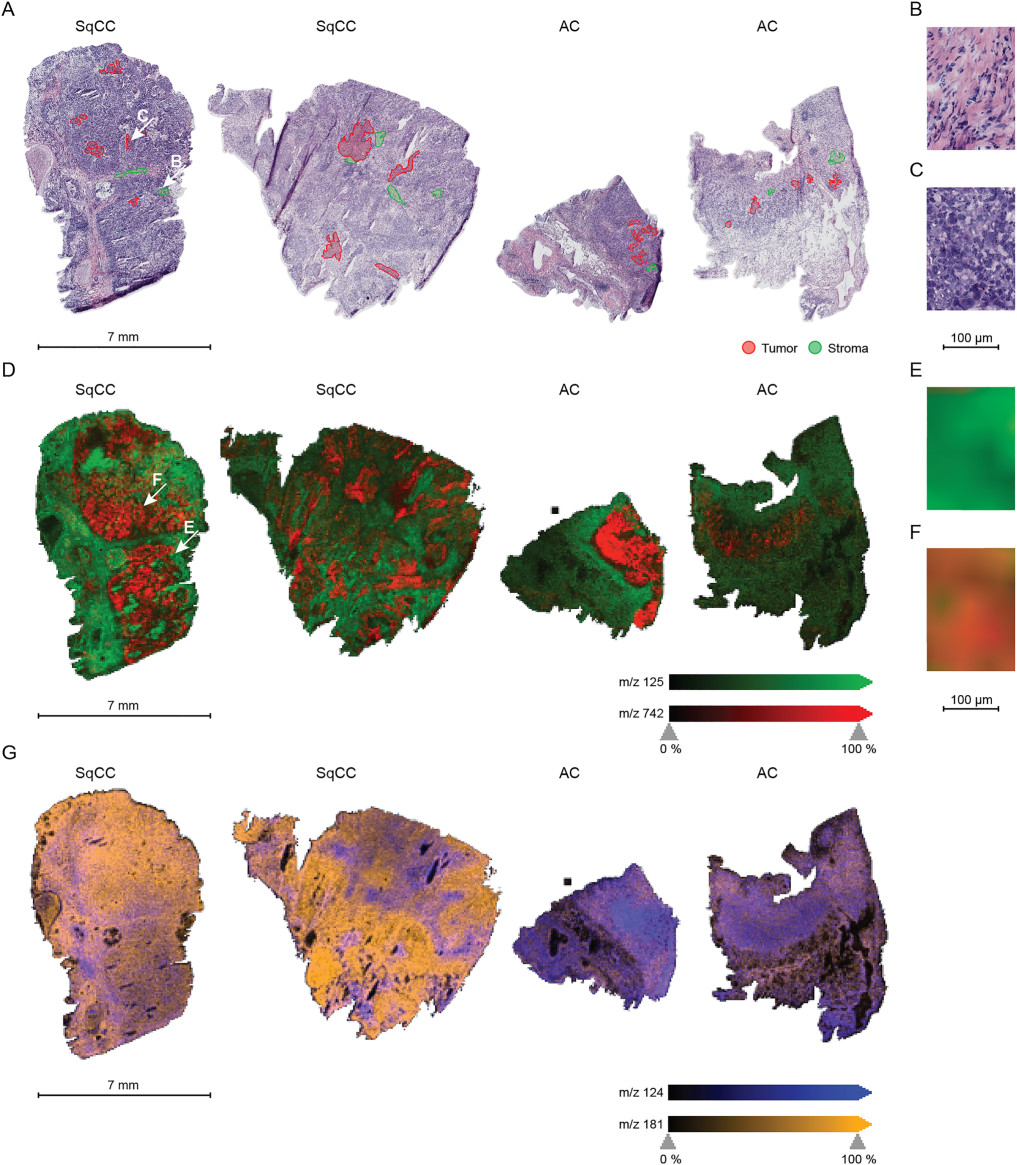
Ion images of selected analytes and corresponding HE images. Four NSCLC cases (SqCC *n* = 2, AC *n* = 2) are shown. **A** HE images with representative annotated regions of tumor (red) and stroma (green). **B** Magnification of stroma region indicated by lower arrow in A. **C** Magnification of tumor region indicated by upper arrow in A. **D** Overlay of ion channels m/z 125 (putatively representing oxalic acid, green) and m/z 742 (phospholipid, red) separates tumor and stroma regions. **E** Magnification of stroma region indicated by lower arrow in D and depicted in B. **F** Magnification of tumor region indicated by upper arrow in D and depicted in C. **G** Overlay of ion channels m/z 124 (representing taurine in purple) and m/z 181 (representing glutamine in orange). Scales are included for overview images (7 mm) and magnifications (100 µm). *HE* hematoxylin and eosin, *NSCLC* non-small cell lung cancer, *SqCC* squamous cell carcinoma, *AC* adenocarcinoma

Spatial distribution of analytes with highest contribution in OPLS-DA are shown in Fig. 3. Representative tumor and stroma regions were annotated in each case, if available (Fig. 3A–C). The differentiation of NSCLC tumor and NSCLC stroma regions is visualized through analytes m/z 125 (green) and m/z 742 (red) (Fig. 3D–F). Metabolic differences between AC and SqCC are depicted in Fig. 3G. While taurine (purple) is abundant in AC tumor regions, the analyte shows lower intensities in SqCC tumor and equal amounts in SqCC stroma. Glutamine (orange) shows higher abundances in SqCC.

### Detection of *IDH* mutations in NSCLC via MALDI-MSI

**Fig. 4:**
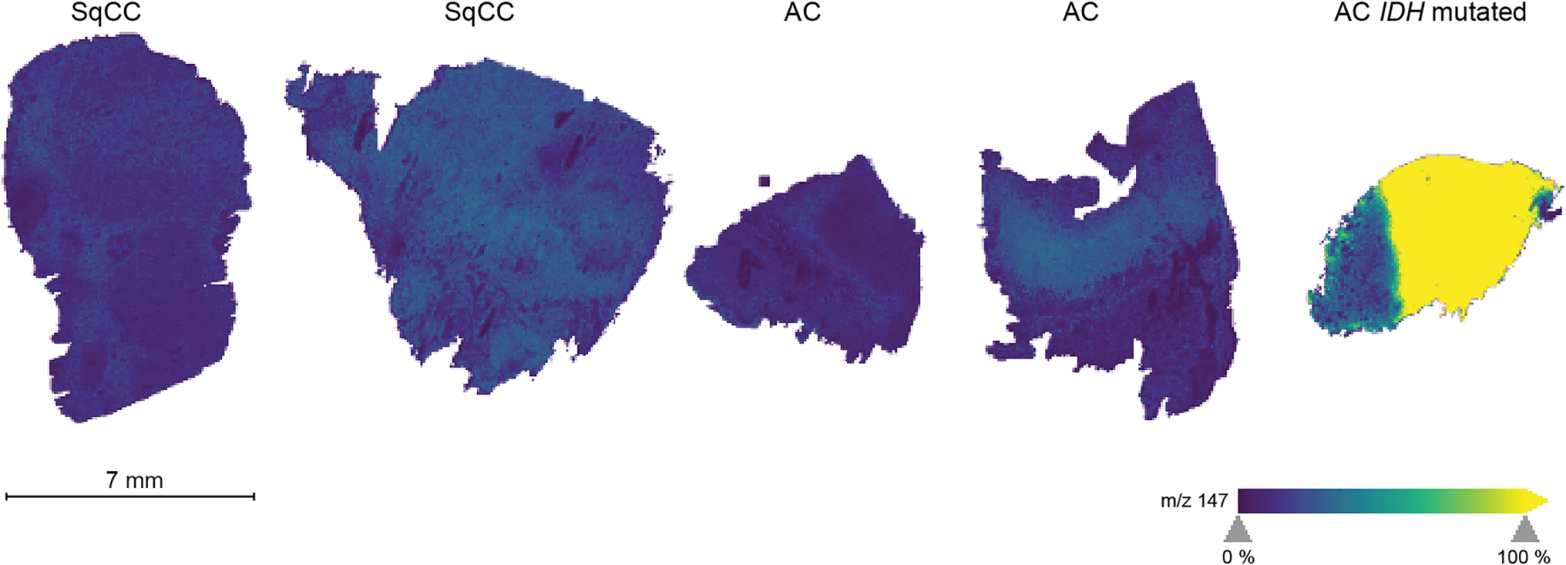
Spatial distribution of 2-hydroxyglutarate (m/z 147) indicates an *IDH* mutation. Enhanced intensities of the oncometabolite are depicted in tumor regions of one sample (far right). Scale (7 mm) is included. *IDH* isocitrate dehydrogenase, *NSCLC* non-small cell lung cancer, *SqCC* squamous cell carcinoma, *AC* adenocarcinoma

One case showed increased intensities at m/z 147 in tumor areas (Fig. 4). This analyte was presumed to be 2-hydroxyglutarate (2HG) which is highly produced as consequence of gain-of-function *IDH* mutations (Dang et al. 2009). The result was reproduced in a measurement with high mass resolving power of an Orbitrap analyzer, whereby 2HG was identified by accurate mass and MS/MS. Subsequently, the case was sequenced, and *IDH1* mutation of c.395G>T or p.R132L was verified. Hence, *IDH*-mutated NSCLC can be clearly distinguished from *IDH* wildtype cases by MALDI-MSI, as detected intensities of oncometabolite 2HG were found to be 45 times higher than the average level in tumor regions. A detailed analysis of the oncometabolite distribution emphasizes tumor heterogeneity. The visualization in situ and an overlay with histopathological results can be found in the supplementary information.

## Discussion

The analysis of NSCLC via histology-guided MALDI-MSI of small molecules revealed differences between tumor and stroma regions as well as between the major NSCLC subtypes AC and SqCC. While immunohistochemical testing is time-consuming, an optimized MALDI-MSI workflow can reveal metabolomic information of tissues in less than 5 min (Basu et al. 2019). Not only speed in diagnosis, but also the ability to be performed on non-fixated tissues makes it most applicable as an additional tool to evaluate samples intraoperatively. Both these feats would allow the technique to be used as an additional diagnostic tool for the evaluation of samples collected through oncological surgery that require intraoperative consultation, since these samples are cut via frozen section procedure anyway. Another advantage is that only a single tissue section is needed for label-free MSI and HE staining, as performed in this study, enabling the analysis of small tissue biopsies. Adding this approach as a possible plausibility check might increase safety in diagnosis and subsequently in treatment.

NSCLC tumor regions were demonstrated to show different metabolomic profiles than corresponding stroma regions in the tumor microenvironment. Classification was achieved with diagnostic ability of 0.96. Phospholipids were found to be severely altered in NSCLC (Marien et al. 2015; Zhang et al. 2019) and consistently contributed to the separation of tumor cells and tumor microenvironment in this study. The lipid interaction of tumor cells and the tumor microenvironment plays a crucial role in cancer progression, as stromal cells can secrete lipids that fuel cancer cells, induce migration, and enhance proliferation of the tumor (Corn et al. 2020).

Diagnostic ability of 0.95 was achieved for subtype classification. Highest discrimination of AC and SqCC tumors was yielded through antioxidant taurine. Supporting this finding, the upregulation of taurine in AC was previously reported through nuclear magnetic resonance spectroscopy (Rocha et al. 2015). Glutamine contributes to the discrimination of subtypes, as well. NSCLC seemingly rather rely on glucose than on glutamine to feed the tricarboxylic acid cycle (Majem et al. 2020). For SqCC, stronger glutaminolytic activity and enhanced reductive carboxylation of glutamine were suggested compared to AC (Rocha et al. 2015; Sellers et al. 2019). Interestingly, both metabolites, taurine and glutamine, were found to be reduced in serum of NSCLC patients (Hu and Sun 2018).

This study has some limitations. Slide-related batch effects were tried to be circumvented by randomly placed samples but have to be kept in mind. Next, markers for NSCLC tumor regions and the subclassification into AC and SqCC have to be confirmed in larger cohorts.

The detection of *IDH*-mutated NSCLC was demonstrated via MALDI-MSI. *IDH* mutations are present in the vast majority of gliomas (Yan et al. 2009) and in approximately 17% of acute myeloid leukemia (Rakheja et al. 2012), though NSCLC with mutated *IDH* are seldom with an occurrence of 0.5–1.1% and only few cases are described yet (Rodriguez et al. 2020; Sequist et al. 2011; Toth et al. 2018). It was recently hypothesized that *IDH* mutations in NSCLC are branching drivers (Rodriguez et al. 2020) and enhance cell proliferation through Fibulin-5 methylation (Yan et al. 2018). In this study, an *IDH*-mutated AC was detected via MALDI-MSI for the first time to our knowledge. Subsequent DNA-sequencing revealed an *IDH1* mutation. Moreover, heterogenous 2HG metabolism was observed with highest oncometabolite concentrations in the tumor center. Patient characteristics coincide with previously published cases as the specimen was subtyped as AC as well as all previously reported cases and harbors a mutation of the cytoplasmic enzyme IDH1 (14 of 19 cases) (Rodriguez et al. 2020; Sequist et al. 2011; Toth et al. 2018). Furthermore, the specimen shows high PD-L1 expression of > 90% as seen in 40% of previously analyzed specimen (> 50% PD-L1) and a *KRAS* driver mutation as the majority of *IDH*-mutated lung AC (Rodriguez et al. 2020; Toth et al. 2018). However, while most patients with *IDH* mutations have an age greater than 70 (Rodriguez et al. 2020; Sequist et al. 2011; Toth et al. 2018), this patient was 57 at resection, being 11.5 years younger than the AC patient median age of the cohort.

Even though occurrence of *IDH* mutations is low in NSCLC, considering the high incidence of this disease and the manifold of developed treatment strategies for *IDH*-mutated cancers emphasizes the importance of further research in this field (Golub et al. 2019; Rodriguez et al. 2020; Toth et al. 2018). Since we were able to reliably detect an *IDH*-mutated specimen within our samples, this approach might enable a cheaper and faster diagnosis and a screening of mutants (e.g. on tissue microarrays) to investigate patient characteristics.

## Conclusions

MALDI-MSI is a powerful tool that could support diagnosis of NSCLC in the future. Given the possibility that new entities can be identified using MALDI-MSI, this might lead to further studies in treatment. The investigation of metabolomic differences between larger cohorts of *IDH*-mutated AC and *IDH*-wildtype AC can support our understanding of this disease. A reliable classification of tumors during a surgical procedure has the potential to improve results and survival of patients after oncological surgery as well as it might enable quicker post-operative care such as chemotherapy or radiotherapy.

## Supplementary Information

Below is the link to the electronic supplementary material.

Supplementary file1 (PDF 418 kb)

## Data Availability

The datasets that were generated and analyzed during the current study are available from the corresponding author HB on reasonable request.
