# Supplementary material for: Subtyping non-small cell lung cancer by histology-guided spatial metabolomics
Source: J Cancer Res Clin Oncol. 2021 Nov 28;148(2):351–60. doi: 10.1007/s00432-021-03834-w (PMC8800912; doi:10.1007/s00432-021-03834-w)
Supplement: Supplementary file 1 — Supplementary file1 (PDF 418 kb) [file 432_2021_3834_MOESM1_ESM.pdf]

Supplementary information: Subtyping non-small cell lung cancer by histology-guided spatial metabolomics: a cohort study

Minor metabolic differences were detected between AC stroma and SqCC stroma as visualized in the receiver operating characteristic (Figure S 1).

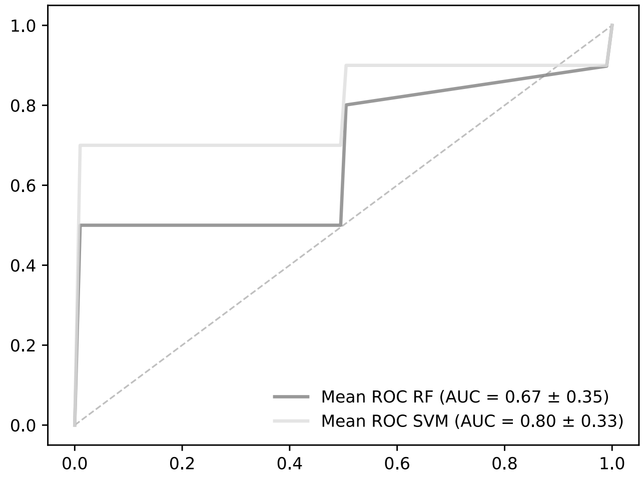

S 1: Receiver operating characteristic describes the diagnostic ability to distinguish adenocarcinoma (AC) stroma from squamous cell carcinoma (SqCC) stroma. Random forest (RF) (dark grey) and support-vector machine (SVM) (light grey) algorithms were utilized. AC (n=17) and SqCC (n=10). Abbreviations: NSCLC: non-small cell lung cancer; SqCC: squamous cell carcinoma, AC: adenocarcinoma, RF: random forest, SVM: support-vector-machine.

Oncometabolite 2-hydroxyglutarate (2HG) shows highest intensities in tumor center (Figure S 2 A). By adjusting the intensity scale, specific abundance of 2HG in whole tumor region is revealed (Figure S 2 B).

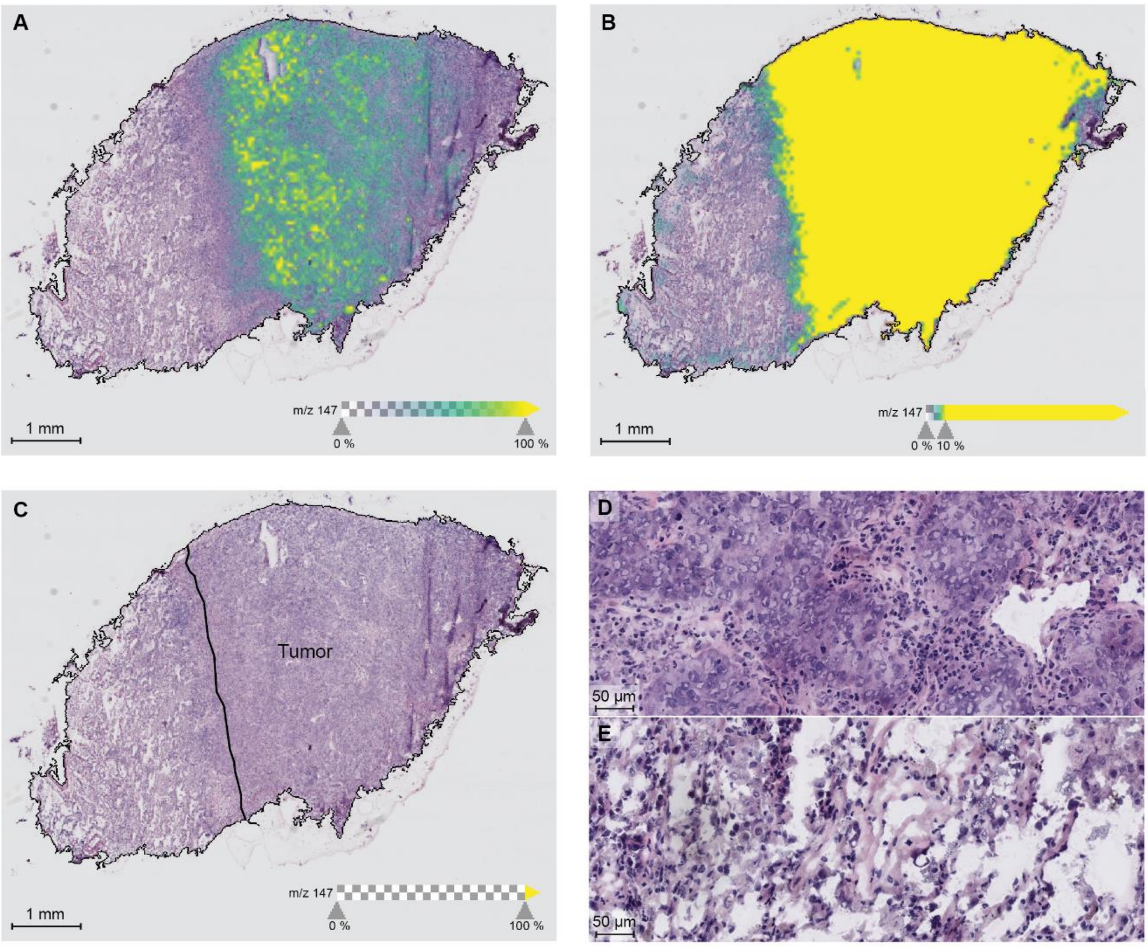

S 2: Distribution of 2-hydroxyglutarate in lung adenocarcinoma with HE overlay. A) Abundance of 2-hydroxyglutarate in tumor region. Scale 1 mm. B) Abundance of 2-hydroxyglutarate in tumor region with adjusted scale (0-10%). Scale 1 mm. C) HE image with tumor annotation. Scale 1 mm. D) HE zoom of tumor region on subsequent section. Scale 50 µm. E) HE zoom of adjacent normal tissue on subsequent section. Scale 50 µm.

The analyte 2-hydroxyglutarate was identified via accurate mass (Table S3) and MS/MS.

S 3: Information on 2-hydroxyglutarate measurement. Detected (TOF and orbitrap) and calculated m/z values, PPM error of detected orbitrap mass, and detected adduct are given.

TOF m/z	Orbitrap m/z	Calculated m/z	Analyte	PPM	Adduct
147.05	147.0293	147.0299	2-hydroxyglutarate	-4.1	$[M-H]^-$

Tables S4 and S5 contain m/z values provided by feature importance. These were used for machine learning algorithms.

S 4: Ion channels used for discriminant analysis of tumor and stroma. Detected (TOF and orbitrap) and calculated m/z values, PPM error of detected orbitrap mass, and detected adduct are given. Analyte name is black if MS/MS validation was performed.

TOF m/z	Orbitrap m/z	Calculated m/z	Analyte	PPM	Adduct
90.03					
121.04	121.0399	121.0407	Nicotinamide	-6.9	$[M-H]^-$
125.02	124.9638	124.9647	Oxalic acid	-7.2	$[M+Cl]^-$
128.06					
150.92	150.9796	150.9804	Fumarate	-5.6	$[M+Cl]^-$
152.92	152.9951	152.996	Succinate	-5.4	$[M+Cl]^-$
190.05					
210.87					
226.84					
267.09					
502.33	502.2965	502.2939	Lysophosphatidylethanolamine	5.2	$[M+Cl]^-$
714.55					
716.57					
738.56					
740.57					
742.59					
743.60					
835.59					
857.59					
859.60					
861.61					
863.65					
887.63					

S 5: Ion channels used for discriminant analysis of subtypes. Detected (TOF and orbitrap) and calculated m/z values, PPM error of detected orbitrap mass, and detected adduct are given. Analyte name is black if MS/MS validation was performed.

TOF m/z	Orbitrap m/z	Calculated m/z	Analyte	PPM	Adduct
106.94					
121.04	121.0399	121.0407	Nicotinamide	-6.9	$[M-H]^-$
124.03	124.0065	124.0074	Taurine	-7.1	$[M-H]^-$
126.02					
128.93					
130.07	130.0501	130.0509	Hydroxyproline	-7.1	$[M-H]^-$
136.05	136.0396	136.0404	Anthranilic acid	-6.2	$[M-H]^-$
145.09	145.06105	145.0619	Glutamine	-5.9	$[M-H]^-$
150.92	150.9796	150.9804	Fumarate	-5.6	$[M+Cl]^-$
154.94					
164.05	164.07117	164.0717	Phenylalanine	-3.2	$[M-H]^-$
169.04					
173.03					
181.08	181.0381	181.0385	Glutamine	-2	$[M+Cl]^-$
183.06					
195.07					
199.06					
216.06					
226.84					
228.84					
273.12					
279.06					
305.09					
307.11					
478.32					
500.31					
501.30					
502.33					
528.31					
714.55					
738.56					
857.59					
863.65					

List of m/z values used for discrimination of subtypes by stroma:

115.04, 117.06, 118.08, 124.03, 137.03, 145.09, 160.01, 166.04, 169.04, 181.08, 183.06, 184.86, 192.05, 195.07, 197.96, 199.06, 199.96, 210.87, 211.03, 215.07, 222.33, 226.84, 248.09, 346.08, 528.31,714.55, 740.57, 773.59, 790.60, 794.63, 835.59, 863.65
